# Increased Temperatures Promote Fruit Enlargement Through Cellular and Transcriptomic Changes in Raspberries (*Rubus idaeus* L.) cv. Heritage

**DOI:** 10.3390/plants15132055

**Published:** 2026-07-02

**Authors:** Jesús Hernández-Urrieta, Sebastián García, Lamia Estait, Francisca Aguilar, José A. O’Brien, Alejandro Jerez, Carolina Contreras

**Affiliations:** 1 Facultad de Ciencias Biológicas, Pontificia Universidad Católica de Chile, Avenida Libertador Bernardo O’Higgins 340, Santiago 8331150, Chile; jmhernandez5@uc.cl (J.H.-U.); lestait0@estudiante.uc.cl (L.E.); jobrieno@uc.cl (J.A.O.); 2 Instituto de Producción y Sanidad Vegetal, Facultad de Ciencias Agrarias y Alimentarias, Universidad Austral de Chile, Isla Teja S/N, Valdivia 5110566, Chile; sebastian.garcia01@alumnos.uach.cl (S.G.); francisca.aguilar@alumnos.uach.cl (F.A.); 3 Departamento de Fruticultura y Enología, Facultad de Agronomía y Sistemas Naturales, Pontificia Universidad Católica de Chile, Vicuña Mackenna 4860, Santiago 7820244, Chile; 4 Laboratorio de Farmacia (Instrumentación Analítica), Instituto de Farmacia, Facultad de Ciencias, Universidad Austral de Chile, Valdivia 5090000, Chile; alejandrojerez@uach.cl

**Keywords:** climate change, quality traits, microscopy, cell cycle, fruit size

## Abstract

Climate change is expected to increase temperatures in agricultural producing regions, potentially affecting fruit development and quality. To date, the molecular responses of raspberry fruits to moderate warming under field conditions have not been explored. In this paper, raspberry plants (*Rubus idaeus* L. cv. Heritage) growing in two contrasting agroclimatic regions of Chile were exposed to a moderate increase in temperature during fruit development. Fruit phenotyping, histological analyses, and RNA sequencing were used to evaluate physiological and transcriptomic responses to warming. Elevated temperature increased fruit weight and fruit dimensions in both orchards and was associated with larger drupelet and cell areas, which was accompanied by reduced cell density. Moreover, transcriptomic analyses revealed marked differences in gene expression responses between raspberries fruits from different locations with only a small number of heat-responsive genes shared across locations. Nevertheless, the common enrichment of oxylipin-related processes was observed, suggesting a conserved response. In addition, a combined treatment model identified the enrichment of processes like ribosome biogenesis, RNA metabolism, cell cycle regulation, cytokinesis, and structural cellular remodeling. These transcriptional changes were consistent with the cellular phenotypes observed in heat-treated fruits. Overall, our results show that moderate warming promotes larger raspberry fruits through changes in cellular organization, while the underlying molecular responses are strongly influenced by agroclimatic context.

## 1. Introduction

In recent years, interest in raspberries (*Rubus idaeus* L.) has increased due to their high nutritional value and health benefits. These fruits are rich in bioactive compounds such as polyphenols, particularly anthocyanins and ellagitannins, which exhibit strong antioxidant and anti-inflammatory properties and are linked to the prevention of chronic diseases [1,2,3,4].

Beyond their nutritional value, raspberries are also appreciated for their sensory attributes, which are determined by a complex composition of primary and specialized metabolites. Fruit quality is strongly influenced by environmental conditions, with temperature playing a key role in plant development, ripening, and metabolism, affecting traits such as sugar–acid balance, firmness, and secondary metabolite accumulation [5].

Rising temperatures due to the climate crisis are expected to alter these processes, posing changes for raspberry fruit quality and flavor perception. Temperature effects on raspberry physiology and metabolism have been previously studied, and the appearance of a white drupelet disorder under extreme heat conditions was documented [6,7]. Likewise, ref. [8] concluded that the area distribution of *R. idaeus* within China will decrease with the climate change mainly due to increase in mean temperature of the coldest quarter and temperature seasonality among other factors. Later, ref. [9] noted that higher temperatures at critical raspberry phenological stages during fruit development altered several quality traits. Recently, ref. [10] found that extreme events such as late spring frosts, excessive rainfall, and extreme heatwaves have a drastic impact on raspberry yield and quality. Despite all these advances, most research has focused on extreme condition simulations or meta-analyses rather than any collection of actual orchard or field data. On the other hand, the effects of moderate nocturnal temperature increases remain understudied, and their impact on plant and fruit physiology is still unclear since it represents a subtle but chronic warming [11].

All these physiological outcomes in the fruit do not necessarily reflect an understanding of the underlying molecular heat responsive mechanisms, particularly at the transcriptomic level, which are highly relevant to elucidating and explaining these mechanisms triggered by climate change. In this regard, transcriptomic approaches have become valuable tools for understanding how plants respond to environmental cues, identifying molecular processes associated with phenotypic adaptation and therefore proposing effective adaptation strategies expected under future scenarios.

Heat stress has been shown to alter the expression of genes involved in growth regulation, metabolism, hormone signaling, oxidative stress responses, and developmental pathways, leading to broad physiological and biochemical changes in raspberry seedlings [12].

At the molecular level, heat responses are coordinated by transcriptional regulatory networks involving heat shock factors (HSFs), MYB transcription factors, and other signaling components that integrate environmental cues with growth and developmental programs [13,14]. For instance, through transcriptomic studies, heat stress has been shown to alter the expression of genes involved in growth regulation, metabolism, hormone signaling, oxidative stress responses, and developmental pathways, leading to broad physiological and biochemical changes in raspberry seedlings [12]. Consequently, transcriptomic approaches offer a powerful strategy to identify candidate genes and regulatory mechanisms associated with fruit responses to warming.

In fruit crops, transcriptomic studies have revealed that elevated temperatures can affect diverse biological processes, including acidity and phenylpropanoid metabolism in grapevine fruits [15], lipid remodeling and aroma related pathways in tomato [16], and stress-associated regulatory networks in raspberry leaves [17]. These observations suggest that fruit responses to heat involve both conserved stress-related mechanisms and species-specific developmental responses.

Despite these advances, little is known about how raspberry fruits respond at the transcriptomic level to elevated temperatures under field conditions or whether such responses are conserved across different agroclimatic regions. Understanding these molecular responses is essential for predicting how future climate scenarios may affect fruit development and quality. Therefore, in this paper, we aimed to characterize the transcriptomic response of raspberry fruit (*Rubus idaeus* L., cv. Heritage) to a moderate temperature increase simulating climate change under field conditions and to assess differences in gene expression between populations grown in contrasting agroclimatic regions in Chile.

## 2. Results

### 2.1. Fruit Quality

To evaluate the impact of higher temperature in fruit development, raspberries were subjected to moderate higher temperature (~4 °C) from fruit set to ripening. Thus, raspberries harvested during the 2023 season and exposed to heat treatment exhibited a significant increase in fruit weight between treatments. Accordingly, polar and equatorial diameters of heat-treated raspberries showed a significant increase when compared to control (CK). That is, heated raspberries were bigger than control fruit (Table 1). Interestingly, there was no significant difference between central and southern orchards for these three quality parameters (weight, polar and equatorial diameters), suggesting a negligible effect of the background temperatures (location) on the fruit size. Fruit firmness, on the other hand, was affected by the orchard location but not the treatment. However, there was a clear effect on the heated raspberries where fruits were softer than the CKs in the central location (Table 1). These results indicate that heat treatment was the main factor affecting the fruit weight and diameter parameters in the raspberries.

In regard to the antioxidant capacity, the related phenolic compounds (DPPH, flavonoid total content and polyphenol total content) showed no significant differences between locations, treatments, or their interactions (Table S1). Interestingly, in all parameters, it was observed that the control fruit showed a lower antioxidant capacity, as well as lower flavonoid and polyphenol contents, than the heat-treated raspberries. Likewise, the northern orchard presented higher values than the southern orchard, suggesting that an antioxidant defense mechanism could have been triggered by the increasing temperatures.

### 2.2. RNA-Seq Output and Overview

To explore the transcriptional changes occurring in response to the heat treatment, we sequenced 12 libraries corresponding to three biological replicates of control and treated pooled fruit samples coming from central and southern locations. Illumina short-read sequencing yielded a total of 1.26 billion raw reads, ranging from 90.2 to 120.1 million per sample and high overall base quality and consistent GC content (45.3 to 45.7%) (Table S2).

**Figure 1:**
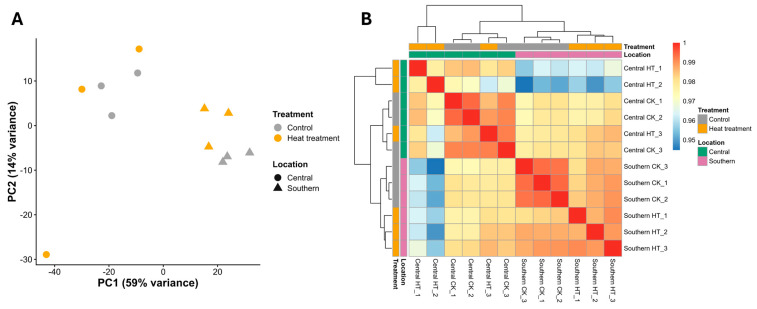
Clustering and similarity of sequencing libraries revealed by PCA and sample correlation. (**A**) Principal component analysis (PCA) of normalized counts for each sequenced library. Different colors represent control (gray) and treated (dark yellow) samples. Triangles represent samples from the southern location and circles from the central location. (**B**) Sample correlation and clustering for each sequenced library. Gray and orange labels represent control and treated samples, respectively. While green labels represent central orchard and pink southern orchard. Blue to red label bar and colors represent a continuous z-score scale of normalized counts per gene. CK stands for control samples, and HT stands for heat-treated samples, while numbers one to three represent biological replicates.

To assess the overall data distribution, principal component analysis (PCA) and sample-to-sample correlation analyses were performed on variance stabilized counts (Figure 1A,B). PCA revealed a clear separation of samples, primarily driven by location along PC1, explaining 62% of the variance, while treatment effects were more subtle and varied between locations (Figure 1A). In particular, the southern location samples showed a clearer separation between control and treated conditions, whereas central orchard samples exhibited a less clear separation. Consistent with this observation, hierarchical clustering based on pairwise sample correlations grouped samples largely by location with the southern orchard replicates forming well-defined clusters according to treatment (Figure 1B). However, treated samples from the central orchard showed reduced within-group similarity with one replicate (Central location Treatment Replicate 3, Central HT_3) displaying increased similarity to control samples relative to the other treated replicates. Based on this pattern, this library was excluded from downstream differential expression analyses.

### 2.3. Differential Gene Expression Analysis Across Locations and Treatments

**Figure 2:**
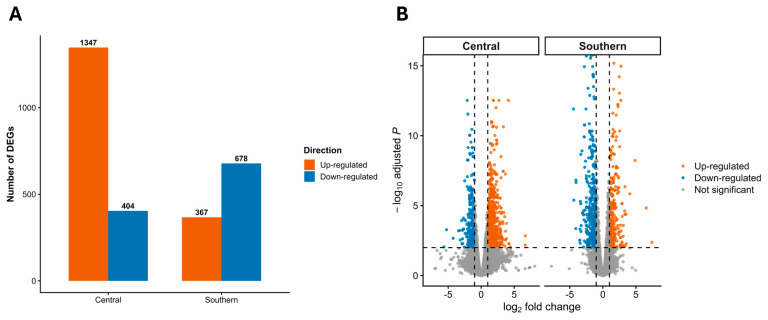
RNA-seq reveals a differential response to a moderate temperature increase between the southern and central orchards. DEGs in the southern and central locations in response to a moderate temperature increase were assessed. Then, the upregulated and downregulated genes were compared between orchards locations according to the number of genes (**A**) and the fold change/adjusted *p* distribution in a volcano plot (**B**). In (**B**) the vertical dashed lines indicate the differential expression threshold (|log_2_FC| ≥ 1), while the horizontal dotted line represents the statistical significance threshold (adjusted *p* < 0.01).

Differential expression analysis revealed distinct transcriptional responses to treatment between locations (Figure 2). In the central location, a total of 1751 genes were differentially expressed with a predominance of upregulated genes (1347 upregulated and 404 downregulated). In contrast, the southern location exhibited a more balanced response, with 1045 differentially expressed genes, including 367 upregulated and 678 downregulated genes (Figure 2A).

These differences were further supported by the distribution of fold changes observed in volcano plots (Figure 2B), where the central location showed a stronger bias toward gene induction, while the southern location displayed a higher proportion of downregulated genes. Together, these results indicate that transcriptional responses to treatment seem to differ between locations both in magnitude and in the balance between gene induction and repression.

### 2.4. Functional Exploration of Shared and Location-Specific Heat-Responsive Genes

**Figure 3:**
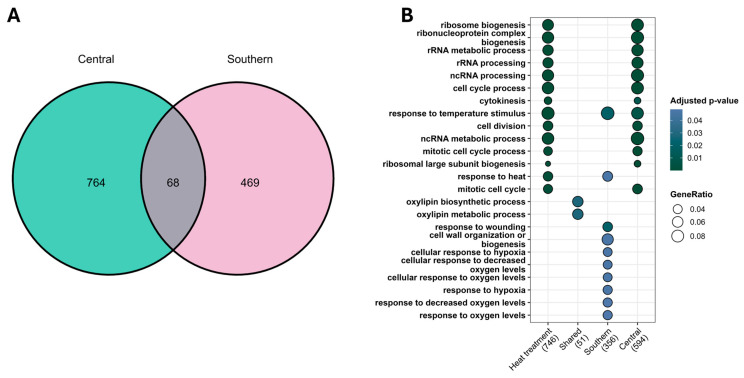
Gene ontology analysis revealed specific and common responses to a moderate temperature increase between the southern and central orchard. (**A**) Venn diagram showing the number of differentially expressed genes (DEGs), based in *A. thaliana orthologs*, specific for the southern and central orchards, and the shared genes between orchards. (**B**) Gene ontology (GO) analysis, specifically biological process (BP), enrichment of the genes in response to a moderate temperature increase in the mixed treatment model (Heat treatment), the shared genes between orchards (Shared), and for the specific genes regulated for the southern and central orchards.

To further explore how transcriptional responses differed between locations, we first examined the overlap between differentially expressed genes (DEGs) identified in the central and southern orchard (Figure 3A). Only 68 genes were shared between both locations, while 764 and 469 genes were uniquely regulated in central and southern orchards, respectively. This indicates that most of the transcriptional response to treatment is location specific with a relatively small set of genes showing consistent behavior across both conditions.

We then performed gene ontology (GO) enrichment analysis using *Arabidopsis thaliana* orthologs to provide functional context for these gene sets (Figure 3B). Shared DEGs were associated with processes such as oxylipin metabolism and response to wounding, suggesting the presence of a common response to treatment. In contrast, the location-specific gene sets showed more varied functional difference with central location-specific DEGs enriched for processes related to ribosome biogenesis, RNA processing, and cell cycle regulation, which is in line with the strong upregulation observed in the central location. Southern location-specific DEGs, on the other hand, were enriched for responses to hypoxia and oxygen-related processes, as well as temperature response genes, pointing to a distinct stress-related transcriptional profile.

To complement this analysis, we also examined genes identified using the combined model, which captures treatment-responsive genes across both locations. This set was enriched for processes such as ribosome biogenesis, RNA metabolism, and cell cycle regulation, reinforcing the idea of a general transcriptional response to treatment. In addition, cellular component enrichment highlighted terms associated with the cell wall, extracellular region, and apoplast, while molecular function categories included microtubule binding, tubulin binding, and motor activity (Supplementary Figures S1 and S2). Together, these results point to coordinated changes in cellular growth, structural organization, and developmental processes in response to heat treatment, which is accordance with the cellular phenotypes, where fruit enlargement is primarily associated with increased cell size and reduced cell density, suggesting a shift toward cell expansion rather than proliferation.

### 2.5. Representative Gene Selection and RT-qPCR Validation

Based on the functional patterns identified in the RNA-seq analysis, we selected a set of representative genes for validation by RT-qPCR. Selection was guided by enriched GO categories, focusing on genes associated with cell cycle regulation (cytokinesis, GO:0000910; cell cycle process, GO:0022402; cell division, GO:0051301; mitotic cell cycle, GO:0000278; mitotic cell cycle process, GO:1903047), cell wall-related processes (plant-type cell wall, GO:0009505; cell wall, GO:0005618), and heat-stress related (response to temperature stimulus, GO:0009266; response to heat, GO:0009408), as well as their expression patterns across locations.

**Figure 4:**
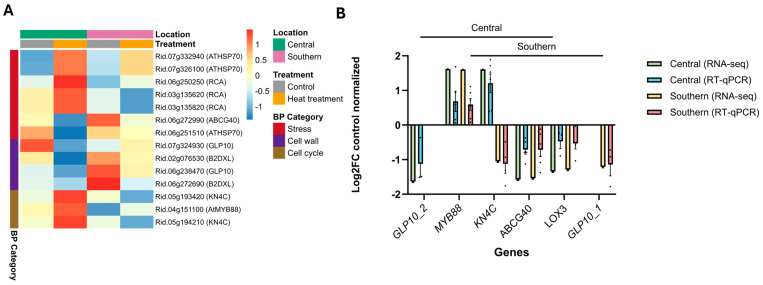
Stress, cell wall, and cell cycle-related genes are differentially regulated in response to a moderate temperature increase between the southern and central location. (**A**) Heat map of normalized counts of selected stress, cell wall and cell cycle-related genes. (**B**) RT-qPCR validation of the cell wall-related genes *GLP10_1* (Rid.06g238470) and *GLP10_2* (Rid.07g324930), together with the cell cycle-related genes *MYB88* (Rid.04g151100) and *KN4C* (Rid.05g193420), oxylipins metabolism related gene *LOX3* (Rid.06g278430) and stress-related gene *ABCG40* (Rid.06g272990), in the southern and central orchards and the comparison with the normalized counts obtained by RNAseq.

To visualize the overall expression patterns of these genes across conditions, we generated a heatmap based on normalized and scaled RNA-seq expression values, including control and treatment samples from both locations (Figure 4A). We observed an increase in expression under treatment in the central orchard across multiple genes, particularly within the cell cycle and stress-related categories, whereas responses in the southern orchard are more variable and, in several cases, reduced or absent.

This resulted in the selection of *RiMYB88*, *RiKN4C*, two *RiGLP10*, *RiABCG40* and *RiLOX3* corresponding to *Arabidopsis thaliana* orthologs previously associated with developmental and stress-related processes. These genes represent key functional categories identified in the enrichment analysis and capture both shared and location-specific transcriptional responses. RT-qPCR analysis was then used to validate these expression patterns (Figure 4B). *RiMYB88*, *RiABCG40* and *RiLOX3* showed consistent expression trends between RNA-seq and RT-qPCR across both locations. In contrast, *RiGLP10* and *RiKN4C* displayed location-dependent responses, which is in agreement with the RNA-seq results. While some variability was observed in the magnitude of expression changes, the overall direction of regulation was largely consistent between approaches, showcasing the robustness of the RNA-seq dataset and reinforcing the presence of both shared and location-specific transcriptional responses to heat treatment, which are also consistent with the phenotypes observed.

### 2.6. Light Microscopy Analysis

To further explore the phenotypic changes observed in fruit size and the possible changes in cell number and cell size suggested by the transcriptomic analysis, the field experiment was repeated in the southern location for the 2025 season, and fruit was collected for phenotypic and microscopy analysis. Thus, the raspberry fruit sampled for microscopy analysis showed the same phenotypic response as the fruit studied for transcriptomic analysis (Table S3). Fruit weight and polar and equatorial diameters were larger in heat-treated raspberries when compared to the control.

**Figure 5:**
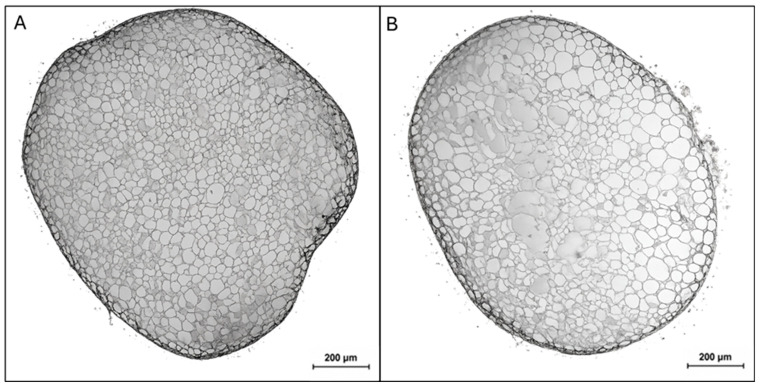
Semithin sections of raspberry drupelet tissue from control (**A**) and increased temperature (**B**) treatments, observed under bright-field light microscopy at 5× magnification. Samples were fixed in Karnovsky solution, embedded in Spurr’s resin, and stained with toluidine blue. Scale bar = 200 µm.

The microscopy analysis carried out in heat-treated and untreated raspberries showed a clear difference in cell number and size (Figure 5). The drupelet area and cell area were larger in the heat-treated fruit than in controls (Table 2). Concomitant with this increase in area, the number of cells in a defined region of interest (ROI) and the cell density were lower in heated raspberries than in controls (Table 2; Figure 5). The lower number of cells, but larger cell area, confirms that the pathways or related genes for cell cycle reported in the transcriptomic analysis for heated raspberries were induced.

**Table 2:** Drupelet and cellular characterization of raspberry fruits cv. Heritage treated and non-treated with increased temperatures from fruit set to ripening (commercial harvest) during the 2025 season for the southern location.

Treatment (T)	Drupelet Area (µm^2^)	Cell Area (µm^2^)	Number of Cells per ROI (n)	Cell Density (Cells µm^−^^2^)
		(3.1 ± 0.5) × 10^3^ b	30 ± 3 a	(1.20 ± 0.08) × 10^−4^ b
Heat	(10.3 ± 0.8) × 10^6^ a	(6.5 ± 0.6) × 10^3^ a	22 ± 4 b	(0.88 ± 0.12) × 10^−4^ a
Significance (T)	*p* < 0.05	*p* < 0.05	*p* < 0.05	*p* < 0.05

Three biological replicates (independent chambers) were analyzed per treatment. One representative drupelet per replicate was used for histological analyses (n = 3). Multiple ROIs (regions of interest) from four semithin sections per drupelet were analyzed for cell quantification. Each ROI was defined as a fixed area of 500 × 500 µm (area = 2.5 × 10^5^ µm^2^). Cell number (n), cell area (µm^2^) and cell density (cells µm^−2^) were quantified within the ROI. Values correspond to mean ± standard error. Different letters indicate significant differences (*p* < 0.05) between treatments for variables showing a significant treatment effect.

## 3. Discussion

The transcriptome analysis and phenotype data were studied in raspberries cv. Heritage that underwent a moderate increased heat treatment simulating potential future conditions caused by climate change to determine whether and how they could affect fruit physiology and the underlying molecular traits. Additionally, this paper was simultaneously carried out in two contrasting geographical locations with different background temperatures to understand how such climate change-derived increased temperatures would impact fruits in the different agroclimatic zones.

We show that increased temperatures during set and ripening resulted in augmented fruit size, as evidenced by higher fruit weight and larger polar and equatorial diameters, and that these changes were independent of the orchards’ geographical location (Table 1). Moreover, upon the microscopical inspection of semithin fruit sections, we determined that this phenotype was explained by a higher drupelet and cell area in heat-treated fruits with respect to control, which was accompanied by a lower number of cells per ROI and overall cell density (Figure 5, Table 2). The inverse pattern between cell size and cell number might be explained by a compensation effect between these two parameters. Likely, this compensation effect is associated with high temperatures at early stages, which reduces cytokinesis. There is evidence that heat affects periclinal division (parallel to the fruit peel) in tomato fruit, especially at early stages of fruit development, whereas anticlinal (perpendicular to the fruit peel) and other type of cells continue cell division for a longer period. Therefore, the duration of cell division is shortened, and the fruit ends up with a lower number of cells globally due to early heating (as was the case in this paper) and with earlier cell expansion rate [16].

This compensation for fruit development at the cellular level under higher temperature regimes has also been reported in tomatoes, along with an increment in fruit size, which also manifests as a compensation effect in fruit growth [18]. The same results were found in melon fruit, where an increased cell size was reported in early stages of development in heated fruit compared to control fruit [19]. Higher temperatures shorten the period of cell division, especially in young fruit period or immature fruit, accelerating fruit development, leading to a lower number of cells, and accelerating cell growth [20].

As for antioxidant capacity and phenolic compounds, they showed no significant differences in this paper. Aguilar et al. [9] reported similar findings with variable results for phenolic compounds and ORAC (antioxidant capacity) in fresh-weight raspberries. Likely, the patterns found in this paper, where the northern orchard and the heat treatments showed higher values for antioxidant capacity and phenolic compounds, were subtle due to the moderate increase in temperature. This suggests that higher temperatures than 4 °C might have had a larger effect, or more replicates (chambers) may have been needed.

While these phenotypic changes were largely consistent between locations (orchards), the underlying transcriptional responses were substantially different. The principal component analysis (Figure 1A) and differential expression analysis showed a strong separation between orchards with only 68 heat-responsive genes shared between locations (Figure 3A) despite the common increase in fruit size under heat treatment. This distinction in the transcriptomic profiles is also present in the predominance of upregulated versus downregulated genes in the central and southern locations, respectively (Figure 2).

In the central location, responsive genes were associated with RNA processing, ribosome biogenesis, cell cycle regulation, and cytoskeleton functions. These categories could be linked to active cellular growth and biosynthetic activity [21], suggesting that elevated temperatures may stimulate developmental and metabolic programs in the fruits. In contrast, genes responsive to heat in the southern location were enriched for responses to hypoxia, oxygen-containing compounds, and temperature-related responses, indicating a transcriptional profile more strongly associated with stress perception and acclimation [22].

Although the origin of such transcriptomic changes remains to be elucidated, they are not totally unexpected, considering that plant transcriptional profiles are highly influenced by environmental conditions [23,24]. In this paper, fruits were produced in distinct agroclimatic regions, which may have contributed to differences in the transcriptional programs in response to warming. Moreover, fruit size, as a quantitative phenotypic trait, is determined by an array of diverse and complex interplays between genetic factors and gene–environment interactions that influence gene expression [25,26]. These characteristics are also usually controlled by robust and to a certain extent, redundant, transcriptional profiles that could allow a single outcome to arise from partially distinct molecular routes.

Additionally, the regulation of gene expression as evidenced by transcript accumulation patterns represents only a single layer among the many that control plant growth and development. Other mechanisms, including alternative splicing, epigenetic regulation, and other post-transcriptional and post-translational control, also respond to environmental conditions and abiotic stress [27,28,29,30] and may therefore contribute to phenotypic convergence beyond what can be resolved from differential gene expression alone.

Furthermore, bulk RNA-seq has also been shown to underestimate the heterogeneity of transcriptional responses at the cellular level, because it tends to average the expression profiles of all cells in the tissue or organ [31,32]. In our raspberry fruits under heat treatment, as well as any other tissues, this means that the transcriptomic profile of less abundant cell types or the expression patterns of lowly expressed but key genes could be masked by overrepresented tissues and transcripts. Consequently, some of the location-specific transcriptional differences observed in this paper could reflect differences in the relative contribution of specific cellular populations rather than distinct biological responses. Future single-cell and spatial transcriptomic approaches could help resolve these limitations and provide a more detailed understanding of how heat influences fruit development across contrasting agroclimatic contexts.

Consistent with the observed enrichment patterns, the selected candidate genes showed expression profiles associated with fruit developmental to heat treatment. While the magnitude of the responses varied between RNA-seq and RT-qPCR, the direction of change was generally maintained. Interestingly, several genes showed contrasting responses between orchards, further illustrating the location dependent nature of the transcriptional response despite the common phenotypic effects observed under heat treatment.

Nevertheless, when looking at the enrichment of the shared genes, we revealed some level of convergence with GO terms related to oxylipins metabolism and biosynthesis. Oxylipins are a diverse class of lipid-derived molecules in plants, which are known to participate in signaling during both abiotic and biotic stress responses [33]. They do so by interacting with other signaling pathways, such as those of auxins, gibberellins, ethylene and abscisic acid (ABA) [34]. Moreover, oxylipin-reactive electrophile species have been shown to induce heat stress-like responses [35], which are associated with thermotolerance [36]. Therefore, the enrichment of these categories suggests that the activation of oxylipin signaling may represent a conserved component of the heat response across orchards even though most downstream transcriptional responses appear to be location specific.

Through both RNA-seq and RT-qPCR, we validated that among the oxylipin metabolism-related genes, *RiLOX3* was downregulated in both locations (Figure 4B), further supporting oxylipin metabolism as part of a conserved response to heat treatment in our design. Its *Arabidopsis* ortholog *AtLOX3* is one the four main 13-lipoxygenases that are known to participate in oxylipin synthesis, including jasmonic acid (JA) [37], and its expression has shown to be regulated by a heat shock factor protein (*AtHSFA2*) [38], which links it to heat responses. Also, a *lox3* loss-of-function mutant was shown to be deficient in JA and other oxylipins [39] in *Arabidopsis*, and the same was found in a Cu*lox3a* mutant in *Cucurbita pepo* [40].

Since oxylipins are known to interact with auxin and gibberellin signaling pathways, which are major regulators of cell expansion and fruit growth, the modulation of *RiLOX3* expression could reflect broader changes in hormone crosstalk occurring under heat. Interestingly, jasmonates are frequently associated with growth restraint and resource allocation toward stress responses [41,42], whereas auxins and gibberellins are generally linked to cell expansion and developmental growth [31,43]. The conserved downregulation of *RiLOX3* observed across both locations raises the possibility that heat may alter the balance between stress-associated oxylipin signaling and growth-promoting hormonal pathways, which could therefore contribute to the increased cell area and reduced cell density observed in heat-treated fruits. Although this interpretation is consistent with both the transcriptomic and cellular observations, the evidence remains correlative, and additional analyses of hormones and oxylipin dynamics will be necessary to determine whether *RiLOX3* directly contributes to the observed cell expansion phenotype.

Beyond the conserved oxylipin-related terms, by employing a combined treatment model, we were able to identify heat-responsive biological processes across orchards (genes that respond to the heat increase independently of the agroclimatic region) that are more directly associated with fruit growth and development. Again, categories related to ribosome biogenesis, RNA and rRNA metabolism, cell cycle regulation and cytokinesis were significantly enriched (Figure 3B), suggesting an affectation of fundamental cellular processes involved in fruit development [44]. Furthermore, cellular component enrichments showed cell wall and apoplast-related terms, while molecular function enrichments showed microtubule organization terms (Figures S1 and S2, respectively), pointing to changes in structural processes within the fruit tissue. The tight coordination of cell cycle control, cell wall and cytoskeleton reorganization have been linked to cell expansion [45,46] and are therefore consistent with the larger cell area and reduced cell density observed in heat-treated fruits.

To further investigate this, we validated the expression patterns of some key genes within these categories through RT-qPCR (Figure 4B), such as *RiMYB88* and *RiGLP10*, which are involved in cell division regulation and cell wall remodeling, respectively. *RiMYB88* is particularly relevant to the observed fruit phenotype, since its *Arabidopsis* ortholog is known to act as direct ‘brakes’ of cell division along with *four lips* (*FLP*), through the repression of the expression of core cell cycle genes, such as *CYCLIN-DEPENDENT KINASE (CDK) B1;1*, *CYCLINA2;3* and *CDKA;1* [47,48]. Moreover, a double loss of function mutants of *myb88 flp* has been shown to undergo repeated mitotic divisions, leading to the formation of clusters of stomata in leaves [49,50]. In the heat-treated fruits, we observed an upregulation of *RiMYB88*, which in the light of what is known about this transcription factor in other species could be associated with a decrease in cell division, helping to explain the observed phenotype (a smaller number of cells and cell density, Table 2). However, whether *RiMYB88* performs a similar function as its ortholog during raspberry fruit development in heat treatment remains to be experimentally determined.

On the other hand, although we did not see a significant effect of heat in fruit firmness, we did see a marked trend toward softer fruits in contrast with the controls (Table 1). To explore this, we looked at the expression by RT-qPCR of cell wall-related genes, particularly two *Rubus idaeus germin-like protein 10* (*RiGLP10*), each of which showed a downregulation specific to one location (Figure 4B). *Glycine max GmGLP10* is localized to the apoplast and plant cell wall, where members of this family have been associated with cell wall remodeling processes, extracellular ROS metabolism, and the maintenance of cell wall integrity [51]. Also, this family of proteins is strongly induced by environmental stressors in different fruits [52]. During fruit development, ripening and response to stress, the structural architecture of the fruit cell wall undergoes dramatic changes and degradation to control texture and softening [53]. Potentially, *RiGLP10s* could then be involved in the firmness modifications observed and would be worth exploring experimentally in the future.

Comparative transcriptomic analyses involving heat-treated fruits remain relatively limited with only a few reported examples showing that response profiles are markedly different among species. For instance, Rienth et al. [15] reported that in grapevine fruit, responsive genes are mainly associated with acidity and phenylpropanoid metabolism, whereas malic acid and anthocyanin-related transcripts were modulated by heat. Almeida et al. [16] found that the transcriptomic data pointed to alterations in lipid metabolism by lipid remodeling affecting membrane fluidity and stability as well as the aroma volatile profile of tomato fruits. In raspberry, our results point toward a combination of stress-associated signaling responses as well as developmental processes related to cell proliferation and expansion, suggesting that indeed, molecular mechanisms underlying fruit responses to heat may be highly species dependent.

## 4. Materials and Methods

### 4.1. Plant Material and Orchard Sampling

Raspberries cv. Heritage were obtained from two commercial orchards (Central and Southern orchard) during the 2023 season. The central orchard was located in the Central Valley of Chile with higher background temperatures and drier climate, whereas the southern orchard was located 850 km south with lower background temperatures and humid climate. The agroclimatic conditions, soil analysis and nutrient composition of each location are found in Aguilar et al. [9].

In each orchard, raspberries underwent 2 treatments: (i) 4 °C of increased temperature (over the ambient temperature) from fruit set to ripening (commercial harvest) and (ii) untreated control (no increased temperature). The heating treatment was imposed on the raspberry primocanes directly on the orchard by using hand-made temperature chambers and controlled heaters to reach 4 °C difference according to the methodology described by [9].

The heat treatment was imposed 11 and 12 days in the central and southern orchard, respectively. Fruit was collected at the red maturity stage from primocanes with similar vigor, hand-picked from the upper third of the canes, between 7 and 8 am to reduce dehydration or any other negative effect derived from radiation and extreme heat. Heat-treated and control fruit were harvested at the same time. The central orchard was harvested on 22 February 2023, while the southern orchard was harvested on 22 March 2023. After harvest, the collected fruit were placed in a 250 g clamshells and transported in coolers equipped with gel pack units to ensure a fruit temperature of 3–4 °C until they reached their destination. Raspberries were transported to the Plant Development and Biotechnology Laboratory at Pontificia Universidad Católica de Chile in the central zone and to the Postharvest Laboratory at Universidad Austral of Chile for the southern orchard.

### 4.2. Fruit Quality Assessments

Twenty raspberries per heating chamber and each control were analyzed. Each fruit was weighed on a digital scale and expressed in g. To estimate fruit size, the equatorial and polar diameters of each fruit were determined using a digital caliper (Kawasaki-chi, Kanagawa prefecture, Japan). Finally, fruit firmness was assessed in a FirmPro equipment (Happy Volt SPA, Santiago, Chile) according to [9] and expressed in gF/mm.

### 4.3. Fruit Antioxidant Capacity Assessments and Phenolic Compounds

The extraction procedures were conducted according to [46]. A 2 g sample was grinded in liquid nitrogen and homogenized with 40 mL of a solvent mixture comprising acetone, water, and acetic acid in a ratio of 70:29.5:0.5 (*v*/*v*/*v*). The resultant mixture was incubated at a controlled temperature of 30 °C for 40 min and then vortexed for 30 s at 10-minute intervals. Thereafter, the mixture was centrifuged at 5000 rpm (equivalent to 4050× *g*) for 10 min at 15 °C. Following centrifugation, the supernatant was subjected to filtration utilizing 125 mm filter paper with a pore size ranging from 20 to 25 µm (designated as code 1238). The volume of the extracted solution was quantified, and the extracts were preserved at −20 °C. These extracted samples were subsequently employed in the quantification of total flavonoids (TFC), total polyphenol content (TPC), and antioxidant capacity assessed through the DPPH methodology. All chemical reagents utilized throughout this experimental protocol, as well as in the DPPH, TPC, and flavonoid analytical procedures, were procured from Merck (Darmstadt, Germany).

#### 4.3.1. Determination of Antioxidant Capacity Using DPPH

The capacity for scavenging the DPPH radical by raspberry extracts was assessed in accordance with the methodology delineated by [54], which is based on the foundational method established by [55]. A modification was introduced at the reference standard; rather than employing ascorbic acid, Trolox was utilized to quantify the free radical scavenging capacity as Trolox equivalents (TEs), adhering to the analytical framework proposed by Enujiugha et al. [56]. In brief, 0.05 mL of the suitably diluted extract sample was combined with 5.0 mL of a methanolic DPPH radical solution. The mixture was vigorously vortexed for 1 min and then at ambient temperature in darkness for 30 min. Thereafter, the absorbance of the sample was quantified utilizing a UV-Vis spectrophotometer at a wavelength of 517 nm against a methanol blank. The negative control was devised by integrating the DPPH solution with 0.05 mL of methanol. Trolox served as the standard reference compound (0–100 mg/L). The antioxidant capacity was expressed as Trolox equivalents (TE) expressed in mg per gram of dry weight.

#### 4.3.2. Total Polyphenol Content (TPC)

The assessment of TPC was carried out according to Singleton and Rossi [57]. A 0.5 mL aliquot of the sample was diluted with 3.75 mL of water, followed by the addition of 0.25 mL of Folin–Ciocalteu reagent, which had been diluted to 50% in an aqueous solution. Subsequently, 0.5 mL of 10% sodium carbonate was incorporated, and the mixture was thoroughly homogenized. The mixture was then allowed to stand for 1 h at room temperature and protected from light exposure. Two blanks were generated following the same protocol, substituting the sample with the extraction solution. Absorbance was recorded at 765 nm using a UV/Visible spectrophotometer. The outcomes were expressed as mg of gallic acid equivalents (GAE) per gram of dry weight (mg GAE/g DW).

#### 4.3.3. Total Flavonoid Content (TFC)

The TFC was obtained with the methodology by Chang et al. [58]. An aliquot of 500 µL of the extract was amalgamated with 1.5 mL of 95% ethanol, 100 µL of 10% aluminum chloride (AlCl_3_), 100 µL of 1 M sodium acetate, and 2.8 mL of distilled water. Following a 30-min incubation period at room temperature, the absorbance of the reaction mixture was assessed at 415 nm utilizing a UV/Vis spectrophotometer (UV-160A, Shimadzu; Kyoto, Japan), employing a standard curve established with quercetin-3-glucoside (5–200 mg/L). The results were articulated as mg of quercetin equivalents (QE) per gram of dry weight (mg QE/g DW). In the current investigation, sodium acetate was utilized in place of potassium acetate as the buffering agent for the medium while maintaining the same molar concentration as delineated in the referenced methodology.

### 4.4. RNA Extraction and Sequencing

A mix of drupelets from five different fruits per condition were ground to a fine powder in liquid nitrogen using a mortar and pestle. Subsequently, the total RNA was extracted from 100 mg of frozen powdered tissue, using the RNAqueous™ Phenol-Free Total RNA Isolation Kit (Thermo Fisher Scientific, Waltham, MA, USA), following the manufacturer’s instructions. After eluding in RNase-free water, samples were snap frozen and kept at −80 °C. Before library preparation and sequencing, the concentration, quality and integrity of the samples were assessed using a Nanodrop 2000 spectrophotometer (Thermo Fisher Scientific, Waltham, MA, USA) and a Fragment Analyzer (Advanced Analytical Technologies, Inc., Ankeny, IA, USA).

For sequencing, a total of 12 libraries (3 control and 3 treatment samples per location) were prepared using a directional mRNA library preparation setting with poly A enrichment to prevent gDNA contamination. Moreover, 150 bp paired-end reads were produced with Illumina NovaSeq X Plus Series technology (San Diego, CA, USA). Library preparation and sequencing were carried out by Novogene Co. (Beijing, China).

### 4.5. RNA-Seq Bioinformatic Analyses

Data were processed as previously described in [32]. Briefly, reads were quality checked using FastQC v. 0.12.1 [59] and trimmed to remove low-quality sequences and remaining adapters using TrimGalore v. 0.6.10 [60]. Reads were then mapped to both Anitra v.1 [61] and JoanJ v.2 [62] *Rubus idaeus* genome assemblies, proceeding with the JoanJ v.2 genome after checking across libraries for mapping percentages and read counts using Rsubread v. 2.14.2 [63] with the respective transcriptome annotation files. Sample-to-sample relationships were assessed using variance stabilized counts from DESeq2 [64], and pairwise sample correlations were calculated and visualized as a heatmap (pheatmap in R, ref. [65]) with hierarchical clustering. Given that RNA was extracted from fruit tissue, which may present inherent biological heterogeneity, replicate consistency was evaluated by comparing within-group and between-group correlations. Samples showing reduced similarity to their corresponding biological group relative to other replicates were considered for exclusion from downstream analyses.

For differential expression analysis, DESeq2 was used to compare both control versus treated samples per location. In addition, a combined model including location and treatment (~location + treatment + location: treatment) was used to identify the overall responsive genes across both locations. In both cases, a threshold of log_2_FC > 1 and an adjusted false discovery rate (FDR) < 0.01 were used to determine differentially expressed genes (DEGs). Prior to functional enrichment analysis, DEGs were converted into *Arabidopsis thaliana* orthologs obtained from the Joan J genome annotation available at the Genome Database for Rosaceae (GDR; https://www.rosaceae.org/Analysis/14031373 accessed on 14 January 2026). Gene ontology (GO) enrichment analysis and visualization were then carried out using clusterProfiler [66], using the biological process, molecular function and cellular component ontology and a Benjamini–Hochberg adjusted *p*-value threshold of 0.05. Candidate genes for validation were selected from DEGs based on functional annotation and GO enrichment results, prioritizing genes representative of key enriched biological processes and exhibiting consistent or contrasting expression patterns across locations.

### 4.6. RT-qPCR

For the validation of RNA-seq results, RT-qPCR analyses were performed using three biological replicates of fruit samples subjected to the same experimental conditions used for RNA-seq, following the previously described protocol for RNA extraction. cDNA was synthesized from 1 μg of total RNA using the All-In-One 5X RT MasterMix (ABM), following the manufacturer’s instructions. Moreover, reactions were carried out using HOT FIREPol^®^ EvaGreen^®^ qPCR Mix Plus (Solis BioDyne, Tartu, Estonia) on an AriaMx Real-Time PCR System (Agilent Technologies, Santa Clara, CA, USA). Each reaction was performed in technical triplicates, and no template controls (NTCs) were included.

Gene specific primers were designed for selected genes: *RiMYB88* (Rid.04g151100), *RiKN4C* (Rid.05g193420), *RiGLP10* (Rid.06g238470 and Rid.07g324930), *RiABCG40* (Rid.06g272990), and *RiLOX3* (Rid.06g278430) corresponding to the *Arabidopsis thaliana* orthologs *MYB88* (AT2G02820), *KN4C* (AT5G60930), *GLP10* (AT3G62020), *ABCG40* (AT1G15520), and *LOX3* (AT1G17420), respectively (Table S4). Quantification was performed using efficiency corrected values by the Pfaffl method (Pfaffl, 2001), which was derived from a linear regression of amplification curves using LinRegPCR [67]. Expression values were normalized using the reference genes *Ri18S* (GenBank, KP125886) [68], *RiEF1a* (Rid.07g311980) and *RiUBC* (Rid.07g332380) [69] and subsequently normalized to the corresponding control condition for each experiment. Final expression values were expressed as log2 fold changes relative to control samples.

### 4.7. Semithin Section Preparation and Light Microscopy Analyss

To confirm the data obtained in the transcriptomic analysis, the same experiment described above with increased temperatures was carried out during the 2025 season in the southern orchard. Hence, five representative fruits (sound and free of defects or physiological disorders) were collected at the red fruit stage from each chamber and control primocanes and immediately transported for microscopy preparation. Raspberry samples were dissected and fixed in Karnovsky’s solution based on the formaldehyde–glutaraldehyde fixation described for electron microscopy [70]. Samples were post-fixed in osmium tetroxide and dehydrated through graded ethanol and acetone series following the plant tissue processing procedures described by Liu [71]. Resin infiltration and embedding were performed using Spurr’s resin, which has been described as suitable for plant tissues in transmission electron microscopy protocols [72]. The complete fixation, dehydration, resin infiltration, and polymerization procedure required approximately 10 days. Semithin sections of ≈500 nm thick were obtained and stained with toluidine blue for bright-field light microscopy observation using a Zeiss Axio Imager Z2 microscope equipped with an HXP epifluorescence system (Oberkochen, Germany). Full processing details, including incubation times and solution ratios, are provided in the Supplementary Material (Table S5).

### 4.8. Experimental Design, and Image Analysis

For the microscopy analysis, three biological replicates (n = 3 per treatment; independent growth chambers), each represented by one drupelet, were analyzed. For each drupelet, four transverse sections were obtained. From these sections, a single region of interest (ROI) was selected per drupelet and used as a technical subsample. ROIs were averaged per drupelet prior to statistical analysis to avoid pseudoreplicates. The ROI (region of interest) was defined as a fixed square area of 500 × 500 µm (area = 2.5 × 10^5^ µm^2^). Within each ROI, the cell number, mean cell area and cell density were quantified. Drupelet area was measured at the whole-tissue level, whereas cellular traits were quantified exclusively within the defined ROI. Data are presented as mean ± standard error. Statistical analyses were performed using one-way analysis of variance (ANOVA) with treatment as a fixed factor. Normality and homoscedasticity assumptions were verified prior to analysis. Differences between treatments were considered statistically significant at *p* < 0.05.

## 5. Conclusions

Elevated temperatures during fruit development increased raspberry fruit size across both orchards primarily through changes in cellular organization characterized by increased cell area and reduced cell density. Climate change will affect fruit size by altering the cell cycle and cell number in raspberry fruits. The impact will be most notable in higher background temperature locations. In lower background temperature locations, climate change will generate gene–temperature responses. This paper’s results emphasize the need to study the differential heat effects of early and late stages of fruit development in future research. In this paper, this effect was not separated in the field treatment; therefore, it is not possible to conclude whether the major findings are triggered at early or late phenological stages. In addition, although the transcriptional responses differed markedly between agroclimatic regions, a conserved oxylipin-associated response was identified together with the common enrichment of biological processes related to ribosome biogenesis, cell cycle regulation, and structural cellular remodeling. These findings suggest that similar developmental outcomes can be achieved through distinct transcriptional strategies depending on the environmental context in which fruits develop.

## Figures and Tables

**Table 1:** Fruit quality parameters of raspberries cv. Heritage treated and non-treated with increased temperatures from fruit set to ripening (commercial harvest) during the 2023 season.

Location	Treatment	Weight (g)	Polar Diameter (mm)	Equatorial Diameter (mm)	Firmness (gF/mm)
Central	CK	1.9 ± 0.06 *	14.4 ± 0.5	14.6 ± 0.4	33.3 ± 1.9
	Heat	2.3 ± 0.04	15.9 ± 0.2	17.0 ± 0.2	26.3 ± 1.2
Southern	CK	2.0 ± 0.02	14.4 ± 0.2	15.0 ± 0.1	25.0 ± 0.3
	Heat	2.3 ± 0.02	15.4 ± 0.2	16.9 ± 0.4	26.3 ± 1.9
Location (L) Treatment (T) L × T		n.s. *p* < 0.0001 *p* < 0.05	n.s. *p* < 0.05 n.s.	n.s. *p* < 0.0001 n.s.	*p* < 0.05 n.s. *p* < 0.05

* Values are means ± SE (n = 6). *p*-values correspond to the effects of location (L), treatment (T), and their interaction (L × T) obtained from a two-way ANOVA. CK indicates control treatment. n.s. indicates non-significant differences.

## Data Availability

The original contributions presented in this study are included in the article/Supplementary Materials. Further inquiries can be directed to the corresponding author.

## Supplementary Materials

The following supporting information can be downloaded at https://www.mdpi.com/article/10.3390/plants15132055/s1, Table S1: Antioxidant activity and phenolic-related compounds of raspberries cv. Heritage treated and non-treated with increased temperatures from fruit set to ripening (commercial harvest) during 2023 season; Table S2: RNA-seq derived per library output and quality parameters; Table S3: Fruit quality parameters of raspberries cv. Heritage treated and non-treated with increased temperatures from fruit set to ripening (commercial harvest) during 2025 season; Table S4: Primers used for RT-qPCR validation and reference genes employed in this paper; Table S5: Stepwise protocol for semithin section preparation of raspberry drupelet tissues following optical microscopy sample processing procedures. The table summarizes the sequence of procedures, reagents, and processing times used during sample preparation; Figure S1: Gene ontology analysis (Molecular Function) revealed specific and common responses to a moderate temperature increase between the southern and central orchard; Figure S2: Gene ontology analysis (Cellular Component) revealed specific responses to a moderate temperature increase between the southern and central orchard. RNA-seq raw data were deposited in the NCBI under BioProject PRJNA1472907.

## Abbreviations

The following abbreviations are used in this manuscript:

ROI	Region of interest
PCA	Principal component analysis
DEGs	Differentially expressed genes
GO	Gene ontology
