# Supplementary material for: Increased Temperatures Promote Fruit Enlargement Through Cellular and Transcriptomic Changes in Raspberries (Rubus idaeus L.) cv. Heritage
Source: Plants (Basel). 2026 Jul 2;15(13):2055. doi: 10.3390/plants15132055 (PMC13364327; doi:10.3390/plants15132055)
Supplement: Supplementary file 1 [file plants-15-02055-s001.zip › plants-4379487-supplementary.pdf]

Supplementary Materials

Table S1: Antioxidant activity and phenolic-related compounds of raspberries cv. Heritage treated and non-treated with increased temperatures from fruit set to ripening (commercial harvest) during 2023 season.

**Effect**		**DPPH (mg TE/g)**	**Flavonoid total content (mg QE/g DW)**	**Polyphenol total content (mg GAE/g DW)**
	Central	9.2 ± 0.6	0.46 ± 0.09	4.5 ± 1.0
Orchard	Southern	8.8 ± 0.2	0.39 ± 0.02	4.1 ± 0.5
Temperature	Heat	9.3 ± 0.4	0.44 ± 0.07	4.4 ± 0.8
	Control	8.6 ± 0.4	0.40 ± 0.04	4.2 ± 0.7
Orchard (O)		n.s.	n.s.	n.s.
Temperature (T)		n.s.	n.s.	n.s.
O X T		n.s.	n.s.	n.s.

* Values are means ± SE. *P*-values correspond to the effects of orchard (O), temperature (T), and their interaction (O × T) obtained from a two-way ANOVA. n.s. indicates non-significant differences.

Table S2: RNA-seq derived per library output and quality parameters.

Sample	Orchard Location	Condition	Biological replicate	Raw reads	Raw data (Gb)	Effective (%)	Error (%)	Q20 (%)	Q30 (%)	GC (%)
CT1	Central (Casablanca)	Treatment	1	98,009,256	14.7	99.14	0.01	99.09	96.52	45.68
CT2	Central (Casablanca)	Treatment	2	120,086,904	18.0	99.10	0.01	99.12	96.59	45.56
CT3	Central (Casablanca)	Treatment	3	102,289,454	15.3	99.06	0.01	99.10	96.53	45.44
CC1	Central (Casablanca)	Control	1	104,512,778	15.7	98.91	0.01	99.10	96.52	45.54
CC2	Central (Casablanca)	Control	2	110,361,810	16.6	99.39	0.01	99.09	96.42	45.33
CC3	Central (Casablanca)	Control	3	92,154,860	13.8	99.01	0.01	99.05	96.34	45.50
PT1	Southern (Paillaco)	Treatment	1	90,197,548	13.5	99.16	0.01	99.10	96.51	45.78
PT2	Southern (Paillaco)	Treatment	2	110,379,480	16.6	98.29	0.01	99.11	96.55	45.64
PT3	Southern (Paillaco)	Treatment	3	112,392,776	16.9	99.20	0.01	99.11	96.55	45.54
PC1	Southern (Paillaco)	Control	1	104,159,754	15.6	98.87	0.01	99.11	96.54	45.65
PC2	Southern (Paillaco)	Control	2	118,583,068	17.8	99.06	0.01	99.10	96.54	45.52
PC3	Southern (Paillaco)	Control	3	101,448,004	15.2	99.16	0.01	99.20	96.72	45.54

Abbreviations:

CC: Casablanca Control (Central orchard),

CT: Casablanca Treatment (Central orchard),

PC: Paillaco Control (Southern orchard),

PT: Paillaco Treatment (Southern orchard),

Table S3: Fruit quality parameters of raspberries cv. Heritage treated and non-treated with increased temperatures from fruit set to ripening (commercial harvest) during 2025 season.

Treatment (T)	Fresh weight (g)	Drupelets (n)	Polar diameter (mm)	Equatorial diameter (mm)
Control	2.9 ± 0.2* b	66.5 ± 0.9 b	12.5 ±0.1 b	14.5 ±0.1 b
Heat	4.2 ± 0.3 a	87.5 ± 1.2 a	13.8 ± 0.7 a	17.6 ±0.4 a
Treatment (T)	< 0.001	< 0.001	p < 0.05	p < 0.05

*** Mean ± standard error. Different letters indicate significant differences ($p < 0.05$) between treatments. Significant p-values ($p < 0.05$) are shown in bold. Three biological replicates (independent chambers) were analyzed per treatment ($n = 3$), each mean value was composed of 20 fruit.**

Table S4: Primers used for RT-qPCR validation and reference genes employed in this study.

Candidate genes for RT-qPCR validation					
Gene	Rubus locus	Arabidopsis ortholog	Function / annotation	Forward primer (5' to 3')	Reverse primer (5' to 3')
RiMYB88	Rid.04g151100	AT2G02820	MYB transcription factor	ATGCAACTTCATACATCAACCCG	TCGTCTTTCCACATTGCCCA
RiKN4C	Rid.05g193420	AT5G60930	Homeobox transcription factor	AGTGCTCCAGGTACCAATGAC	CAGGCTCCTGTAGCAGATCG
RiGLP10_1	Rid.06g238470	AT3G62020	Germin-like protein	AGAACGAAGCAAACGCAACC	CAATTCGGGCCAAAGAGACG
RiGLP10_2	Rid.07g324930	AT3G62020	Germin-like protein	CTTTGGCCACCTCCAAAGGA	GCTGGCTTTTGTTGCCGTAA
RiABCG40	Rid.06g272990	AT1G15520	High affinity abscisic acid (ABA) transporter	TTGGGGTTACTGGATGTCGC	TTGTTGAGTCCGGCAGTACC
RiLOX3	Rid.06g278430	AT1G17420	LIPOXYGENASE 3	AAGAAGGGTACTGTGGCTGC	AAATGTTTGGCAACGGCCTC
RiUBC	Rid.07g332380		Reference gene used for normalization	AGGGAATCCCACCGGACCAG	TCAGCCAAAGTGCGACCATCC
RiEF1A	Rid.07g311980		Reference gene used for normalization	AGGAGCCCAAGTTCTTGAAGA	CCTCACAGCAAAACGACCAA
Ri18S rRNA	KP125886 (NCBI)		Reference gene used for normalization	CTACCTATTGTAAGGAATGGTGCCT	TTCTGCATCCGAGATATCAAGTAGT

Table S5: Stepwise protocol for semithin section preparation of raspberry drupelet tissues following optical microscopy sample processing procedures. The table summarizes the sequence of procedures, reagents, and processing times used during sample preparation.

Day	Step	Procedure	Reagent / Solution	Duration
Day 1	1	Primary fixation	Karnovsky solution (4% paraformaldehyde + 2.5% glutaraldehyde in 0.1 M cacodylate buffer, pH 7.2)	24 h
Day 2	2	Storage after fixation	Karnovsky solution	Until processing
Day 2	3	Buffer washes	0.1 M cacodylate buffer (pH 7.2)	3 × 30 min
Day 2	4	Post-fixation	1% osmium tetroxide	2 h
Day 2	5	Water washes	Distilled water	3 × 30 min
Day 2	6	Ethanol dehydration I	10%, 20%, 30%, 50%, 70% ethanol	1 h each
Day 2–3	7	Overnight dehydration	70% ethanol	Overnight
Day 3	8	Ethanol dehydration II	80%, 96%, 100% ethanol	1 h each
Day 3	9	Final ethanol dehydration	100% ethanol	2 changes
Day 3	10	Acetone exchange	Acetone	2 × 45 min
Day 3–8	11	Resin infiltration	Acetone:Spurr resin (7:1, 6:2, 5:3, 4:4, 3:5, 2:6)	Overnight each step
Day 8	12	Pure resin infiltration	Spurr resin	8 h
Day 9	13	Polymerization	Spurr resin	24 h
Day 10	14	Semithin sectioning	—	—
Day 10	15	Semithin staining	Toluidine blue	Routine staining
Day 10	16	Light microscopy observation	Bright-field microscope	—

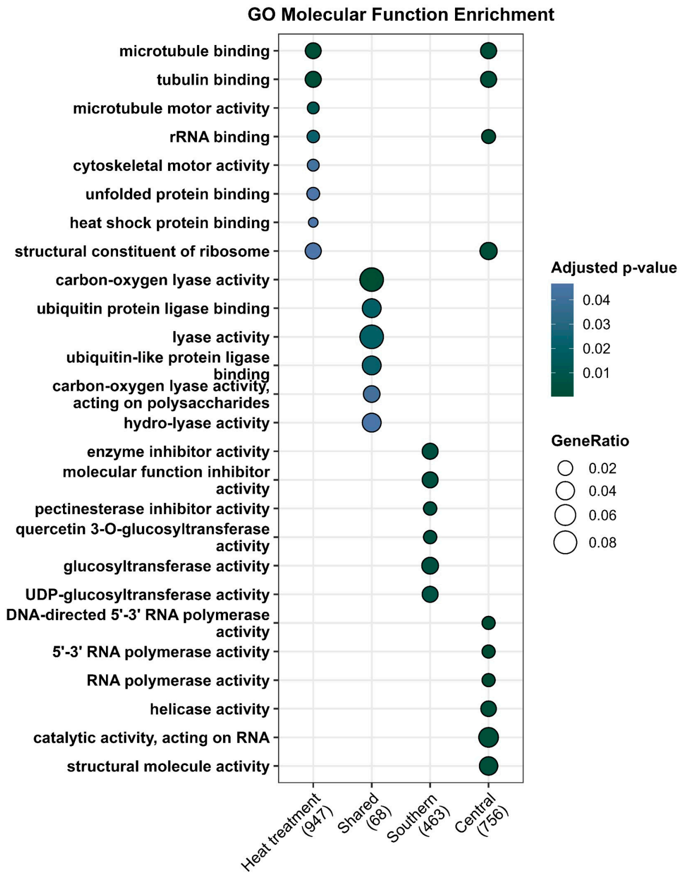

Figure S1: Gene ontology analysis (Molecular Function) revealed specific and common responses to a moderate temperature increase between the southern and central orchard

Gene ontology (GO) analysis, specifically Molecular Function (MF), enrichment of the genes in response to a moderate temperature increase mixed treatment model (Heat treatment), the shared genes between orchards (shared), and for the specific genes regulated for the southern and central orchards.

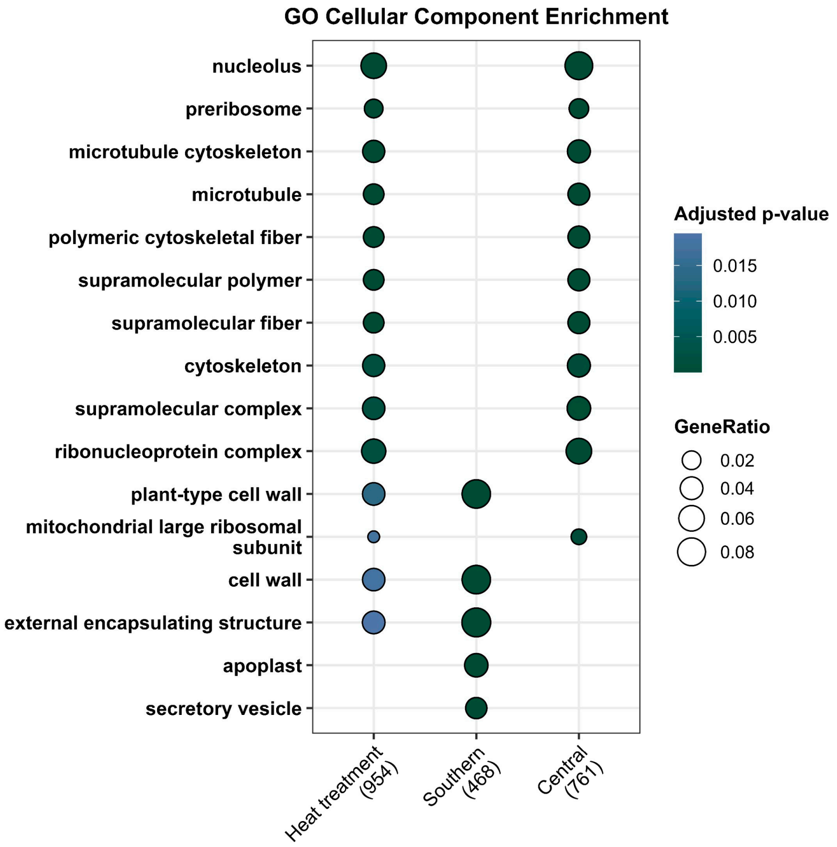

Figure S2: Gene ontology analysis (Cellular Component) revealed specific responses to a moderate temperature increase between the southern and central orchard

Gene ontology (GO) analysis, specifically Cellular Component (CC), enrichment of the genes in response to a moderate temperature increase mixed treatment model (Heat treatment), and for the specific genes regulated for the southern and central orchards.
